# Migrations of cancer cells through the lens of phylogenetic biogeography

**DOI:** 10.1038/s41598-021-96215-9

**Published:** 2021-08-25

**Authors:** Antonia Chroni, Sayaka Miura, Olumide Oladeinde, Vivian Aly, Sudhir Kumar

**Affiliations:** 1 Institute for Genomics and Evolutionary Medicine, Temple University, Philadelphia, PA 19122 USA; 2 Department of Biology, Temple University, Philadelphia, PA 19122 USA; 3 Center for Excellence in Genome Medicine and Research, King Abdulaziz University, Jeddah, 21589 Saudi Arabia

**Keywords:** Cancer, Biogeography, Cancer, Evolution

## Abstract

Malignant cells leave their initial tumor of growth and disperse to other tissues to form metastases. Dispersals also occur in nature when individuals in a population migrate from their area of origin to colonize other habitats. In cancer, phylogenetic biogeography is concerned with the source and trajectory of cell movements. We examine the suitability of primary features of organismal biogeography, including genetic diversification, dispersal, extinction, vicariance, and founder effects, to describe and reconstruct clone migration events among tumors. We used computer-simulated data to compare fits of seven biogeographic models and evaluate models’ performance in clone migration reconstruction. Models considering founder effects and dispersals were often better fit for the clone phylogenetic patterns, especially for polyclonal seeding and reseeding of metastases. However, simpler biogeographic models produced more accurate estimates of cell migration histories. Analyses of empirical datasets of basal-like breast cancer had model fits consistent with the patterns seen in the analysis of computer-simulated datasets. Our analyses reveal the powers and pitfalls of biogeographic models for modeling and inferring clone migration histories using tumor genome variation data. We conclude that the principles of molecular evolution and organismal biogeography are useful in these endeavors but that the available models and methods need to be applied judiciously.

## Introduction

Cancer is a product of somatic evolution. Tumors arise from somatic mutations that accumulate over time, resulting in significant genetic heterogeneity seen in next-generation sequencing surveys^1–4^. This genetic heterogeneity has become key information for inferring clone phylogenies and migration histories^5–9^. In addition to mutations, cancer cells' continuous movements also modulate inter- and intra-tumor diversity, enabling the reconstruction of founder cancer cell genomes and their evolutionary trajectory over time and space in a patient. Retrospective inferences of cancer cells’ evolutionary relationships and spatial dynamics have become the holy grail of understanding tumor evolution and metastasis.

**Figure. 1:**
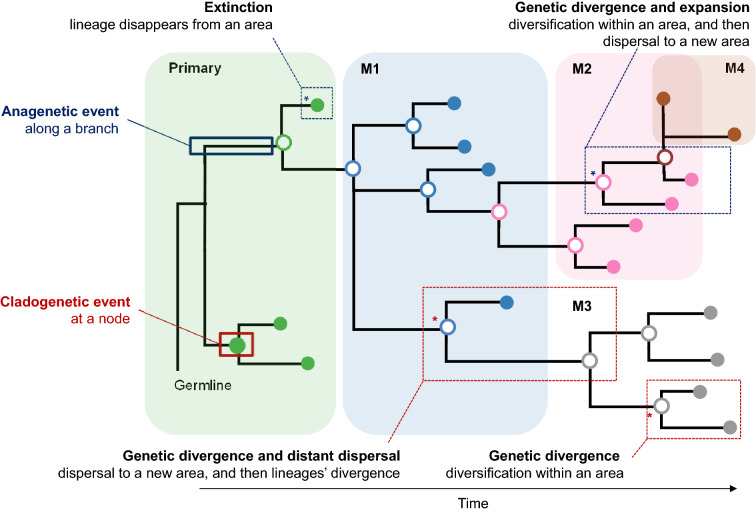
A phylogeny traces the origin and trajectory of movements (anagenetic events in dark blue) and pinpoints diversification and speciation events (cladogenetic events in red). Biogeographic processes (dashed boxes) are exemplified into the presented clone phylogeny of one primary and four metastases: (i) genetic divergence: diversification within an area, (ii) extinction: lineage disappears from an area, (iii) genetic divergence and expansion: diversification within an area, and then dispersal to a new area, and (iv) genetic divergence and distant dispersal: dispersal to a new area, and then lineages’ divergence. Tumor clones are colored based on the source of tumor site: primary (green), and metastases M1 (blue), M2 (pink), M3 (gray), and M4 (brown).

Traditionally, molecular phylogenies have been used to reconstruct the evolutionary relationships of species. A phylogeny is also useful to trace the origin and past geographic distribution of species and populations, including diversification and divergence events. This investigation field is known as phylogenetic biogeography^10–12^. Similarly, a clone phylogeny depicts evolutionary relationships of cancer cells in a patient, revealing genetic divergences and extinctions of cancer cells in a patient. It also provides direct insights into the history and spatiotemporal trajectories of clones across tumors (Fig. 1). Recently, tumor biogeography has been introduced to reconstruct events of cancer cell migrations in tumors^8,9^.

In this article, we apply and test seven biogeographic models, including various parameters described above, in inferring clone migration seeding events and paths using computer-simulated and clinical data. Existent studies and methods showcase the value of evolutionary and spatial frameworks in inferring tumor clone migration histories^5,7–9^. Here, we have mapped organismal biogeographic processes (genetic divergence, extinction, expansion/dispersal, and founder events) explicitly to cancer cell dynamics and evaluated their relative usefulness in reconstructing cancer cell migration histories. Thus, we examined whether the use of biogeographic models, considering diversification, dispersal, extinction, and founder events, would produce better results than previous phylogenetic approaches designed for tumor data that do not model such events. We also explored the suitability of different biogeographic models and evaluated the impact of different biogeographic processes on inferring clone migration events. We analyzed simulated datasets that have been previously used in several studies to benchmark the performance of the biogeographic methods to infer cancer cell migration paths^5,7,9^. Their use permits us to compare results obtained from biogeography methods explored here with the inferences afforded by complementary statistical and algorithmic approaches^5,7^. Our results reveal factors to consider when applying biogeographic models for inferring cancer cell migration paths. We also show how real-case scenarios can benefit from biogeographic models by reconstructing clone migration paths of patients with basal-like breast cancer using published clone phylogenies^13^.

## Results

### Conceptualization of organismal to tumor biogeography through clone phylogenies

We first map organismal biogeographic concepts and models to the process of migration and colonization of cancer cells during metastasis. Tumors are populations consisting of a diversity of cancer cells with different genetic profiles that may represent different lineages in the clone phylogeny. We use the example in Fig. 1, which contains a phylogeny of 17 clones found in one primary tumor (P) and four metastases (M1–M4). Events occurring along a branch in a phylogeny are anagenetic events, which include diversification, extinction, and expansion^12,14^. In organismal evolutionary biology, anagenetic events are not directly observed except through the fossil record. However, one can map the collection of genetic variants that likely arose on individual lineage in a phylogeny. In many cancers, sequencing of temporally sampled biopsies' can directly reveal anagenetic events similar to the sequencing of ancient DNA in paleogenomics.

The other types of evolutionary events in the phylogeny are cladogenetic, including genetic divergences and dispersals (Fig. 1). Genetic differences observed among species and populations are the key to detect cladogenetic events reconstructed in molecular phylogenies of living descendants. In cancer, temporal sampling of biopsies can reveal cladogenetic events that produced extinct descendants.

In biogeography, genetic divergence results in the diversification of lineages within an area. Sometimes, the term duplication is used, but we avoid its further use because of the confusion it may cause in evolutionary genomics. Divergence events are also observed in a clone phylogeny, particularly when clone lineages diverge from each other within a tumor or across tumors. The exact opposite of genetic diversification can also be observed when lineages partially or fully disappear from the phylogeny. Extinction can occur due to random chance, selection, or environmental pressures. Even though extinction is rarely discussed in tumor clone phylogenetics, it happens frequently.

Phylogenies also reveal movements of lineages between locations (geographic areas or body parts) when the locations of individual cells, species, or populations are known^5–9,15^. When lineages accumulate genetic differences along a branch in the phylogeny, and the evolved lineages migrate to a new area, we observe an expansion event. Expansions differ from dispersals in such that the growth of a population occurs in the same place. This movement of cells of a clone from one location to another, where they would potentially form a metastasis, results in the dispersal of  these cells of that clone to additional areas, which is modeled by a dispersal rate (*d*) in organismal biogeography. When a clone genetically diverges following its migration, then a distant dispersal event is said to have occurred. Similarly, when a clone diverges from the rest of the clones within a tumor and disperses to another tumor, we have observed an expansion event. Thus, clone phylogenies can give insights into the origin and trajectory of cancer cells between tumors.

When a clone is no longer present at a location, it is extinct at that location. Extinctions are modeled by an extinction rate (*e*) in biogeographic models. As a result of extinction, the range of descendent clones on a phylogeny can be smaller than the ancestors. Biogeography models also have a parameter (*J*) to consider founder events that establish new populations from a few individuals. In phylogenies, founder events can be detected if only one or a few cells are found to have moved from one location to another to start diversifying in a new area. Both distant dispersal and founder events may result in forming a new colony of cells, i.e., a new metastasis in the case of cancer cell migrations. The primary distinction between dispersal and founder events is the relative number of migrating cells. Founder events are due to one or a few cells, whereas dispersal events involve a larger number of migrating cells. Founder events are expected to be more common in tumor evolution because metastases are thought to be formed by the spread of only one or a few cancer cells. These biogeographic events have been mathematically modeled and implemented in various approaches to infer species migration events^12^, which are directly applicable in the inference of cancer cell migrations between tumors.

### Model fits

We began by analyzing the statistical fits of six biogeographic models (Table 1) to 80 computer-simulated tumor evolutionary datasets. Simulations enable us to assess the performance of computational approaches and reveal potential caveats associated with their use because the ground truth is known. These datasets were simulated using four main clone migration schemes defined by the different number of migrating clones (1–3), the small and large number of tumor areas (5–7 tumors, m5 datasets; 8–11 tumors, m8 datasets), and the different types of source areas of migration (primary or metastasis). The following seeding scenarios reflect this complexity of the clone migration schemes: monoclonal single-source seeding (mS), polyclonal single-source seeding (pS), polyclonal multisource seeding (pM), and polyclonal reseeding (pR) (see “Methods” section).

**Table 1:** Phylogenetic and biogeographic events considered in seven biogeographic models used for analysis.

Phylogenetic events	Biogeographic events	Parameters in biogeographic models						
		BBM	BAYAREALIKE	BAYAREALIKE + J	DEC	DEC + J	DIVALIKE	DIVALIKE + J
Anagenesis (along a branch)	Expansion	Yes^a^	Yes	Yes	Yes	Yes	Yes	Yes
	Extinction	Yes^a^	Yes	Yes	Yes	Yes	Yes	Yes
Cladogenesis (at a node)	Genetic divergence	Yes^a^	No	No	Yes	Yes	Yes	Yes
	Distant dispersal	Yes^a^	Yes	Yes	Yes	Yes	Yes	Yes
	Vicariance	No	No	No	Yes	Yes	Yes	Yes
n/a	Founder-event effect	No	No	Yes	No	Yes	No	Yes
n/a	Descendant range size	1	≥ 2	≥ 2	≥ 2	≥ 2	≥ 2	≥ 2
n/a	Distance-dependent effect on the dispersal probability	No	Customize	Customize	No	No	No	No
n/a	Time	Customize	Customize	Customize	Customize	Customize	No	No

Expansion (dispersal), extinction, and founder events are modeled through *d*, *e*, and *J* parameters, respectively. BBM infers ancestral ranges with biogeographic processes being annotated in RASP (marked as ^a^).

We considered biogeographic models that weigh genetic divergence, dispersal/expansion, and extinction events differently (Table 1). We also explored the provision of including founder events in our models on the accuracy of detecting clone migrations. The parameterization of the aforementioned events results in models with two free parameters, i.e., dispersal rate (*d*) and extinction rate (*e*), and models with three free parameters by adding the founder-event speciation (*J*); see “Methods” section for more details.

**Figure 2:**
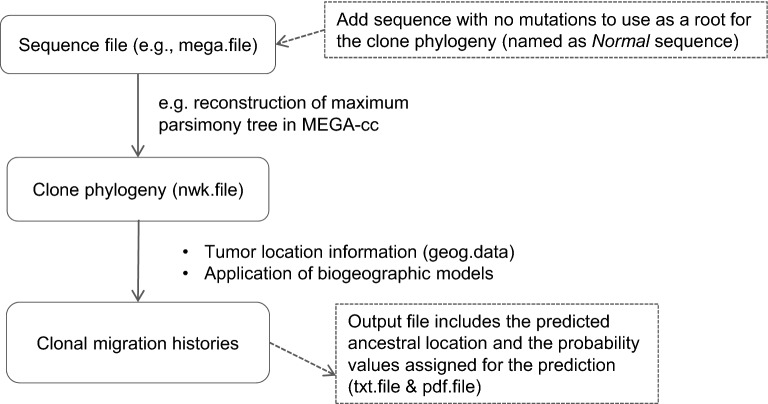
Data analysis pipeline using BioGeoBEARS in R^14^ to infer clonal migration histories.

Overall, we tested six biogeographic models for their fit to the tumor data, three models with two free parameters and three others with three free parameters. BAYAREALIKE, DEC, and DIVALIKE models have two parameters each. They are nested within their respective models that add the founder effect, resulting in a model with three free parameters (hereinafter +J models). We used the BioGeoBEARS software for all model fit analyses. In data analysis, we first inferred phylogeny of cancer cell populations (clone phylogeny) using an existing method^16^, followed by the use of BioGeoBEARS to infer the clone migration history in which the clone phylogeny is used along with the location of tumor sites in which each clone is observed (Fig. 2). BioGeoBEARS estimates the probabilities of annotating internal nodes with tumor locations. These annotations are then used to derive cancer cell migration paths when two adjacent nodes are annotated with different tumor locations. In these analyses, we assumed the correct clone phylogeny because our focus was not assessing the impact of errors in a phylogeny on the accuracy of clone migration inferences. We also compared the accuracy of migration histories reconstructed using biogeographic models in BioGeoBEARS with those obtained from the approaches that do not model biogeographic processes (BBM^9^, MACHINA^5^, and PathFinder^7^).

We first conducted Likelihood Ratio Tests (LRTs) to examine the improvement offered by considering founder events in modeling tumor migrations. In this case, the fit of the BAYAREALIKE, DEC, and DIVALIKE models was compared to their +J counterparts, respectively. The null hypothesis was rejected for more than 50% of the datasets (BAYAREALIKE: 71.25%, DEC: 60%, and DIVALIKE: 53.75%; *P* < 0.05), which means that the model with founder-event effects provided a better fit for a majority of datasets. Interestingly, results differed between BAYAREALIKE and other models (DEC and DIVALIKE) for smaller datasets. For DEC and DIVALIKE, the null hypothesis was rejected in only 20–40% of the datasets with low complexity compared to 60–90% of the ones with more complicated migration graphs (Table 2). For BAYAREALIKE, the null hypothesis was rejected for more than 50% of the datasets in almost all seeding scenarios (except for the m5pM, which was 20%; Table 2). This indicates that a more complex model fits the data better than a simpler model.

**Table 2:** The biogeographic model fits for 80 simulated datasets.

Tumor sites	Seeding scenario	LRTs %/BIC			Best-AICc%					
		BAYAREALIKE/+J	DEC/ + J	DIVALIKE/+J	BAYAREALIKE	BAYAREALIKE + J	DEC	DEC + J	DIVALIKE	DIVALIKE + J
m5	mS	80/20	40/60	30/0	20	40	40	0	0	0
	pS	50/50	30/50	20/0	20	0	50	0	30	0
	pM	20/10	20/20	20/0	10	10	20	0	60	0
	pR	80/80	70/80	80/0	0	30	30	40	0	0
m8	mS	80/20	90/100	60/0	20	50	0	20	10	0
	pS	80/20	60/80	60/0	20	30	20	30	0	0
	pM	90/20	80/90	80/0	10	30	0	30	0	30
	pR	90/40	90/100	80/0	10	20	0	60	0	10
Overall		71	60	54	14	26	20	23	13	5

Percent datasets in which the Likelihood Ratio Test (LRT) rejects the null hypothesis (LRTs%), and in which the model has the best AICc score are shown (Best-AICc%).

Second, we compared the fits of these three non-nested models by using the small-sample size corrected Akaike Information Criterion (AICc). AICc suggested that BAYAREALIKE + J and DEC + J models received the best AICc scores (26.25% and 22.5% of the datasets, respectively) (Table 2). We observed that DEC and DIVALIKE fitted better for simple seeding scenarios and the small number of tumors. In contrast, BAYAREALIKE had a more consistent performance across different complexities in seeding scenarios and different numbers of tumor sites. Estimation of Bayesian Information Criterion (BIC) resulted in similar patterns predicting that more complex models fit the data the best, except for DIVALIKE and DIVALIKE + J.

Our analyses show that models accommodating founder events fit tumor sequencing data better, especially when many tumors were sampled. Still, we observed that more sophisticated models (+J) did not fit better for datasets with a small number of tumor sites and in the presence of reseeding.

### Estimation of ranges at ancestral nodes

In organismal biogeography, researchers often expect and explore the hypothesis of vicariance, i.e., the genetic divergence of a population due to geographic isolation typically caused by a physical barrier. Vicariance causes the split of an area into multiple ones, a type of biogeographic process that is not reasonable when examining cancer cells' movements in a tumor site. Vicariance events are incorporated in the biogeographic models examined. This showed as inference of multiple ranges at ancestral nodes. To better understand this,  suppose for example clones *a* and *b* are sampled in tumors A and B, respectively. In that case, there are three ancestral range possibilities: two are dispersals from areas A and/or B, and one is vicariance from area AB.

**Figure 3:**
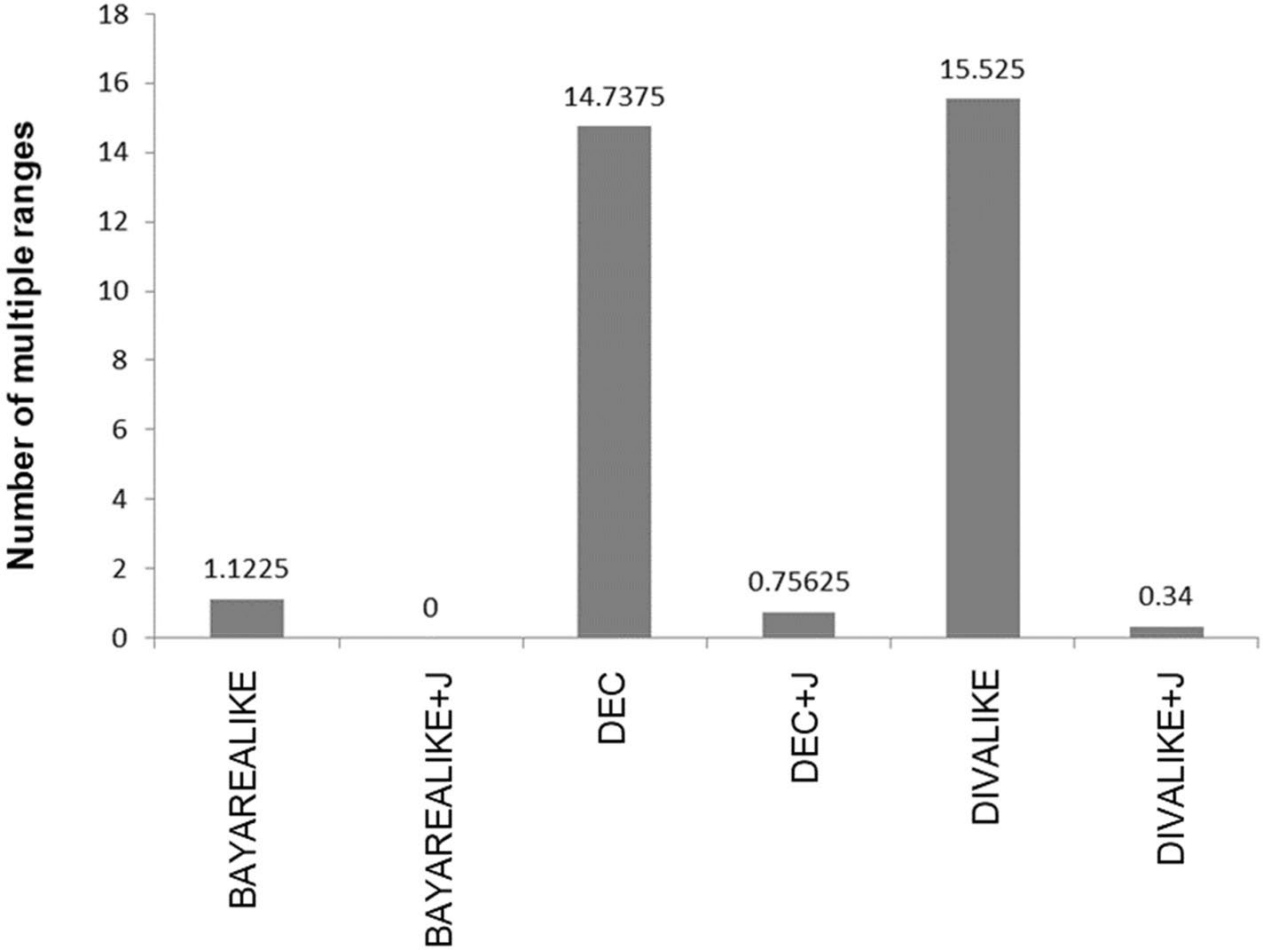
The average number of multiple ranges suggested at ancestral nodes differed among the two and three free parameter biogeographic models.

In our data analysis, five of the biogeographic models, except for BAYAREALIKE + J, predicted multiple ranges at ancestral nodes of the clone phylogeny instead of a single ancestor area (Fig. 3). DEC and DIVALIKE models predicted many multiple ranges (average of 14.74 and 15.53, respectively). These models prefer vicariance, so they end up inferring multiple ranges at the ancestral nodes to explain clone divergences and movements. However, vicariance is unlikely to be a major force in tumor clonal biogeography because of the lack of a physical medium in the same anatomical site that would not allow the dispersal of cancer cells within an area. Therefore, the use of DEC and DIVALIKE may result in erroneous inferences of clone migrations. In fact, this problem is remedied in analysis in which the founder effect is considered. DEC + J and DIVALIKE + J predict less than one multiple range (0.76 and 0.34, respectively; Fig. 3). The average number of multiple ranges was low for the BAYAREALIKE model (average of 1.12), and it became zero in BAYAREALIKE + J.

### Accuracy of migration paths inferred with biogeographic models

**Figure 4:**
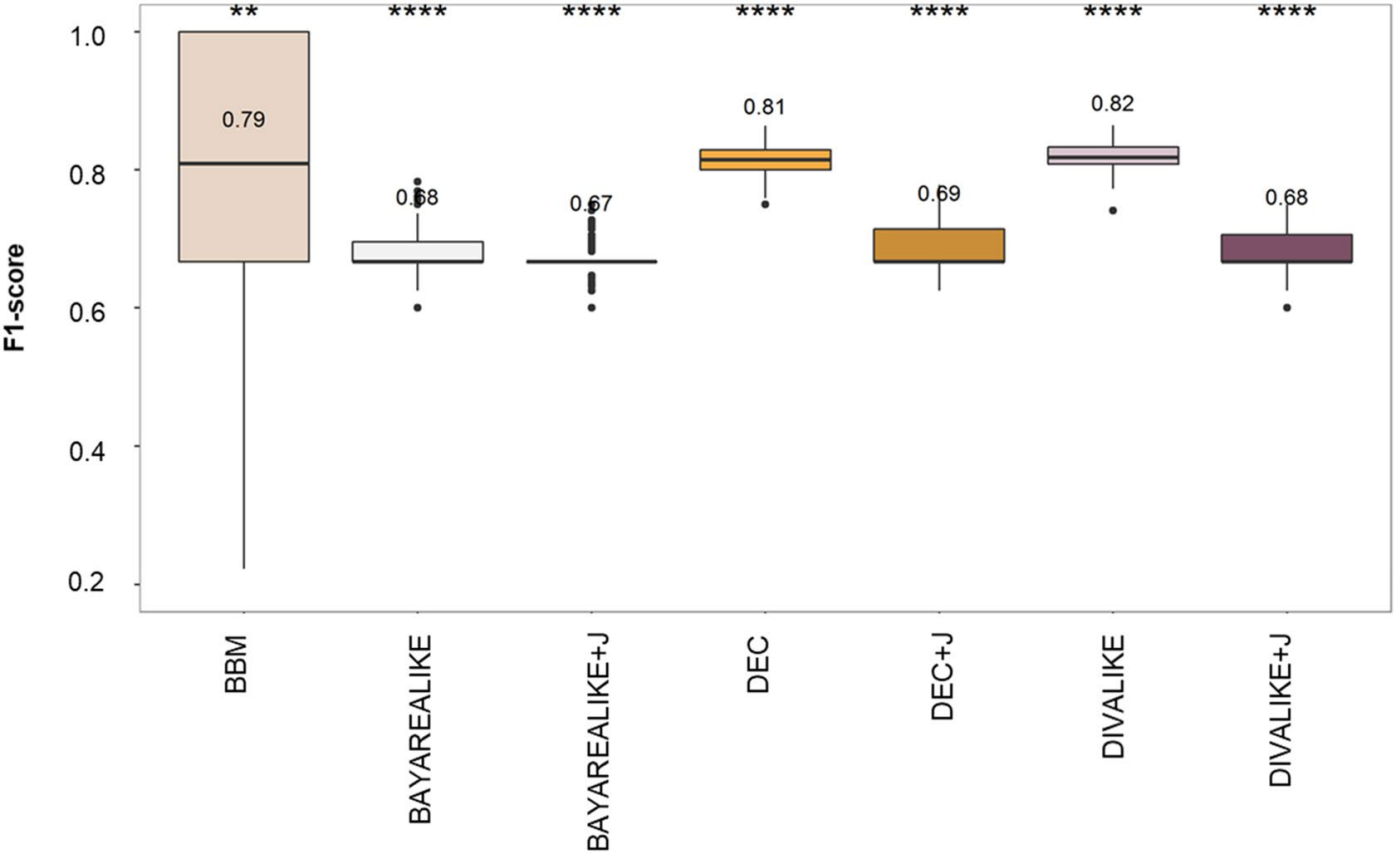
Performance of the seven biogeographic models used for clone migration inferences as measured by F1-scores. Mean values are depicted above the box plots. Differences in F1-scores were examined through a *t*-test and are marked when significant.

We assessed the accuracy of clone migration inferences by using F1-scores (Fig. 4). The F1-score considers true positive (correct), false positive (wrong), and false negative (missing) inference of migration paths. In our evaluations, these represent correct, erroneous, and not-inferred clone migration paths.

Overall, the highest average F1-score was 0.82 for DIVALIKE, followed by DEC and BBM (0.81 and 0.79, respectively; Fig. 4). All other models produced lower accuracy (0.67–0.69). Although the differences in mean F1-scores between BBM and the best method (DIVALIKE) were statistically significant, only BBM produced perfect inferences (F1 equal to 1.0) for some datasets. The best-fitting model (BAYAREALIKE + J) performed significantly worse than some others (0.67). The +J versions of the three models performed significantly worse, with DEC and DIVALIKE producing more accurate migration paths than DEC + J and DIVALIKE + J. There was no difference between the performance of BAYAREALIKE and BAYAREALIKE + J. Therefore, the best-fitting model was identified due to over-parameterization. The use of +J will not improve the accuracy of the migration inferences in tumor data analysis. Furthermore, the method that does not use model biogeographic processes (BBM) was among the most accurate. Also, other methods without modeling of biogeographic processes (MACHINA^5^ and PathFinder^7^) were previously reported to perform similar or better than BBM for inferring migration events of cancer cells^9^.

**Figure 5:**
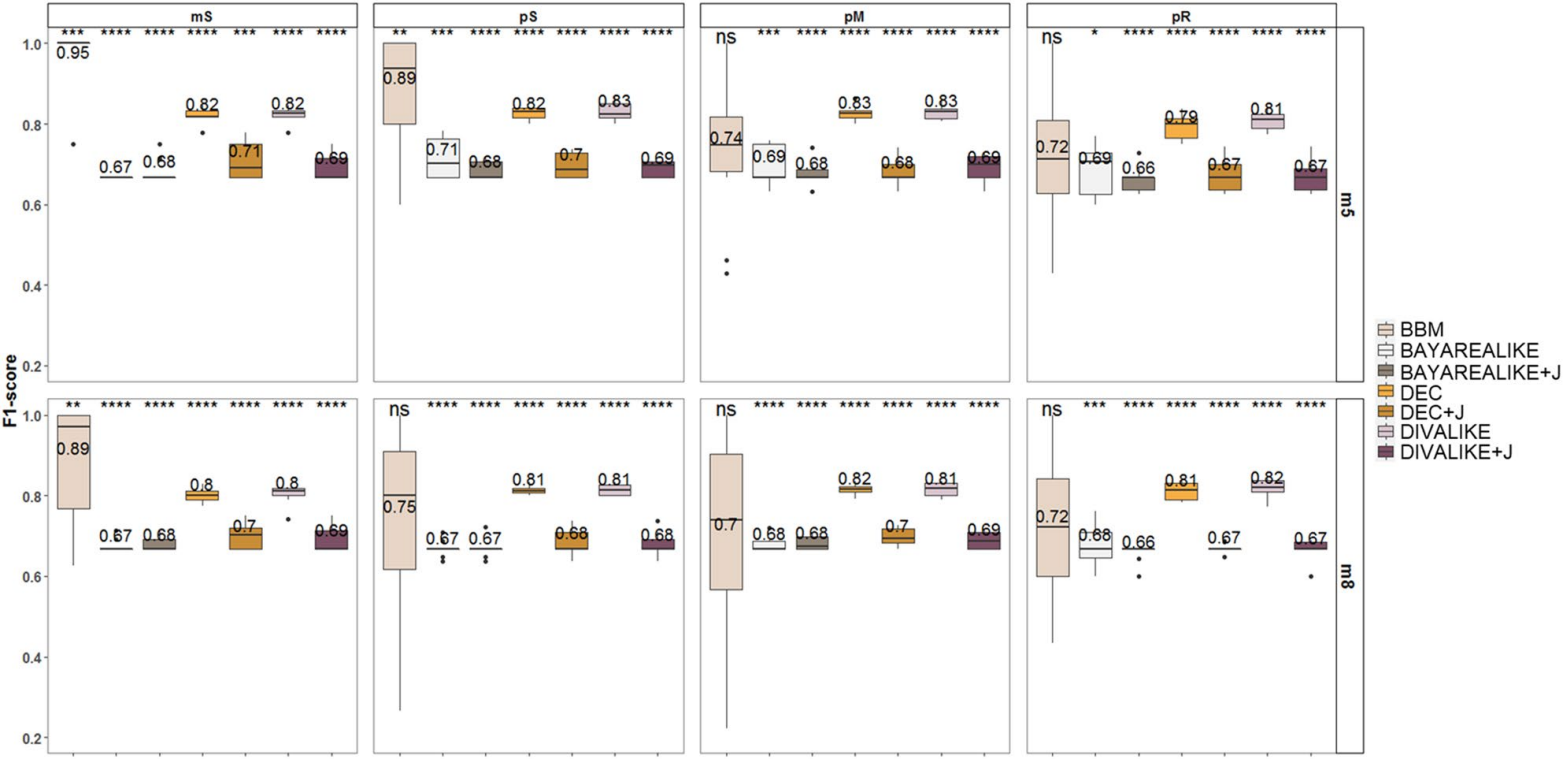
Performance of the seven biogeographic models in inferring clone migrations as measured by F1-scores. Mean values are shown above the box plots. Accuracies are shown based on the number of tumors within datasets (5–7 tumors for m5 and 8–11 tumors for m8) and on the complexity of migration schemes within datasets (monoclonal single- source seeding, mS; polyclonal single-source seeding, pS; polyclonal multisource seeding, pM; polyclonal reseeding, pR). Differences in values of F1-score on tumor count and seeding scenario were examined through t-test and are marked when significant (*, **, ***, ****).

To further dissect the accuracy differences, we examined the effect of the number of tumors (m5 and m8 datasets) and the complexity of the seeding scenario (mS, pS, pM, and pR) on the clone migration inferences (Fig. 5). F1-scores were slightly worse for datasets with higher than those with a small number of tumors (m5; 0.66–0.95, and m8; 0.66–0.89).

F1-scores for most complex seeding scenarios (pM and pR; 0.70–0.72) were worse than single-source seeding (0.75–0.95) in the BBM model. The number of tumors seems to dictate the accuracy of clone migration inferences by BBM in the simplest data (mS and pS), but not in most complex ones (pM and pR). That is not the case for the rest of the models, as their performances remain poor but steady across complexities of metastatic migrations.

**Figure 6:**
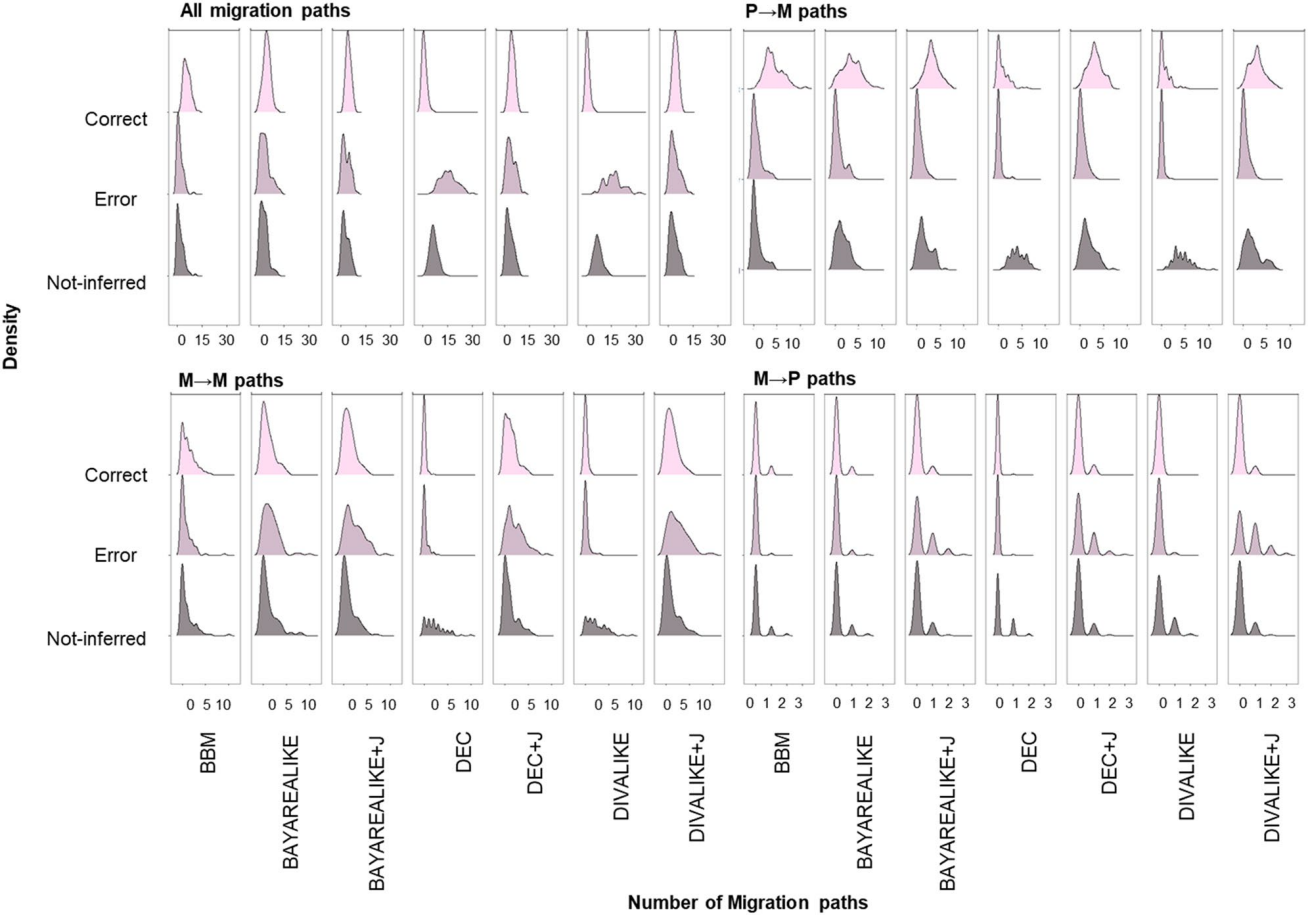
Correct, erroneous, and not-inferred migration paths inferred using seven biogeographic models. Here, we show the results depending on the source-area of the migration: all migration paths, primary to metastasis (P → M), metastasis to metastasis (M → M), and metastasis to primary (M → P).

Next, we examined the numbers of correct, erroneous, and not-inferred migration paths depending on the source- and recipient-area of the migration for each biogeographic method used. In the graphs presented in Fig. 6, density plots show the number of the datasets (y-axis) in which the inferred number of migration paths were observed (x-axis). Four sets of plots are shown: (i) all migration paths; (ii) primary to metastasis paths, P → M; (iii) metastasis to metastasis paths, M → M; and (iv) metastasis to primary paths, M → P. Within each panel, three graphs are included to display the propensity of a given method to infer correct paths (top), erroneous paths (middle), and paths not-inferred (bottom).

For example, in the graph showing all the migration paths for the BBM model, we observe that many correct migration paths were reconstructed for many datasets. Furthermore, there are only a few wrong paths inferred for a smaller number of datasets. A similar pattern was seen for true paths not-inferred (Fig. 6). For P → M migrations, many biogeographic models performed equally well, with a few notable trends. BBM showed a smaller tendency not to infer correct P → M paths. DEC and DIVALIKE reconstructed a smaller number of correct and incorrect paths compared to other methods, resulting in a larger number of datasets with many P → M paths remaining undetected. All biogeographic models failed to detect many M → M paths. BBM, DEC, and DIVALIKE produced fewer wrong M → M paths. Still, DEC and DIVALIKE inferred fewer correct migration paths as well. Interestingly, +J models produce more correct and erroneous M → P paths than their counterparts without the J parameter. However, BBM produced fewer wrong and not-inferred paths, making it the most accurate model.

### Biogeographic analyses of breast cancer metastases

**Figure 7:**
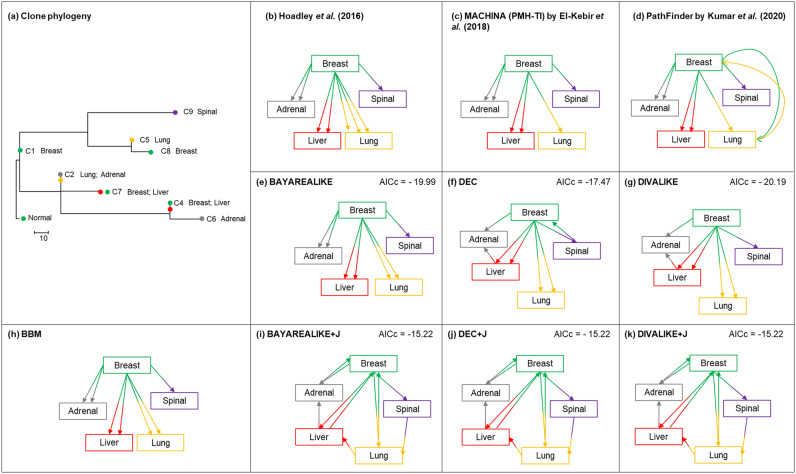
Analysis of Patient A1 with basal-like breast cancer^13^. (**a**) Clone phylogeny and tumor location of each clone reported in the original study. (**b**–**k)** Clone migration histories predicted by non-biogeographic and biogeographic models. Colors correspond to the tumor location where clones were sampled from. Values on the top right are AICcs.

**Figure 8:**
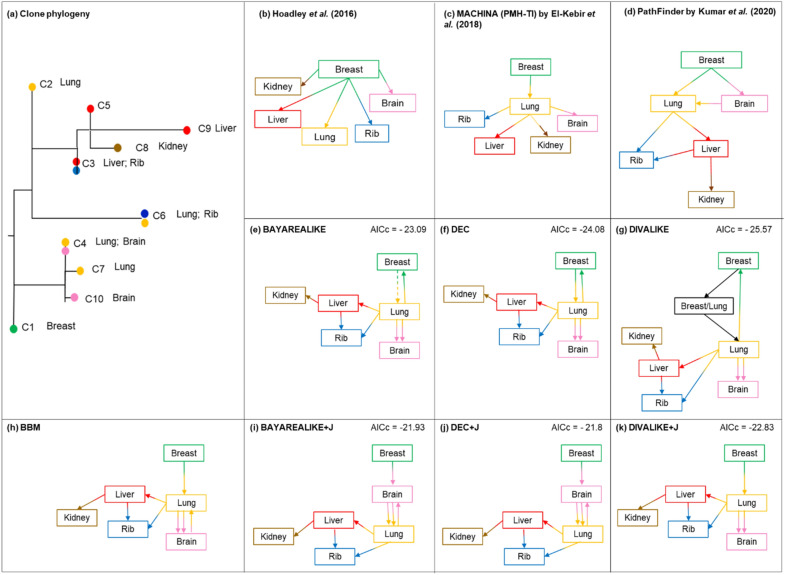
Analysis of Patient A7 with basal-like breast cancer^13^. (**a**) Clone phylogeny and tumor location of each clone reported in the original study. (**b**–**k**) Clone migration histories predicted by non-biogeographic and biogeographic models. Colors correspond to the tumor location where clones were sampled from. Values on the top right are AICcs. Clone migration history inferred by BAYAREALIKE (panel **e**) predicted the origin of metastasis to be from lung tissue, but the estimated probability value of lung at the root of the tree was very low and similar to that for breast tissue (primary tumor). We show the migration history starting from breast tissue (dotted line) because the primary tumor was found there (**e**).

We inferred clone migration histories for two patients (A1 and A7) with basal-like breast cancer^13^ (Figs. 7 and 8, respectively). We begin with patient A1’s dataset consisting of nine clones from one primary tumor (breast) and four metastases (adrenal, lung, spinal, and liver). The phylogeny and tumor sampling locations are shown in Fig. 7a.

For the A1 patient, Hoadley et al.^13^ suggested that clones from the primary tumor seeded all the metastases^13^. Similar to the analysis of computer-simulated datasets, +J models fit better in the Likelihood Ratio Tests comparing BAYAREALIKE, DEC, and DIVALIKE with their +J counterparts, respectively (2∆*ln*L/BIC of 4.5/5.5, 9.5/10.5, and 10/10.9, respectively; *P* < 0.05) (Fig. 7). The use of the BAYAREALIKE model suggested that clones from the primary tumor seeded all metastases. More than one primary clone seeded some metastases (clones C4 and C7, Fig. 7a). This pattern of a primary-to-metastasis spread is similar to that inferred by Hoadley et al.^13^ and by methods that do not involve biogeographic modeling (BBM, PathFinder, and MACHINA; Fig. 7b–d). Importantly, however, not all model combinations and methods produce identical migration history, which was similar to the trends observed in the analysis of computer-simulated datasets.

The second dataset analyzed came from patient A7, which consisted of ten clones sampled from one primary (breast) and five metastatic tumors (brain, lung, rib, liver, and kidney). Figure 8a shows the phylogenetic relationship of clones and their sampling sites of patient A7. Hoadley et al.^13^ suggested that the breast's primary tumor directly seeded all the metastases for this patient^13^. While different types of migration graphs were reconstructed by the biogeograhic models, none of them predicted a migration history in which the primary tumor seeded all the metastases, contrary to Hoadley et al.^13^ suggestion (Fig. 8). Again, models with +J fit the data the best, consistent with the results seen in the analysis of the computer-simulated datasets (2ΔlnL/BIC of 4.6/5.6, 2.3/3.3, and 5.5/6.5, respectively; P < 0.05).

All biogeographic models predicted that metastases seeded other metastases much more frequently than the primary tumors in patient A7 (Fig. 8e–k). In all migration history reconstructions, one or more clones migrated between metastases. Similarly, methods that do not consider biogeographic models (BBM, MACHINA, and PathFinder) predicted many migration paths between metastatic tumor sites. For example, there were two migration events from the lung to brain metastases and one reseeding event from the brain to the lung when BBM was used (Fig. 8h). Most of the methods showed slightly different clone migration histories from each other. However, the seeding of kidney metastasis by the liver metastasis was inferred in all analyses, and the rib metastasis received clones from both liver and lung metastases. The lung metastasis also contributed clones to the brain metastasis. Some biogeographic models predicted reseeding of the primary tumor (breast) by metastases, but this pattern was not universally observed. Overall, non-biogeographic models agree with the type of migration patterns inferred by the biogeographic models indicating metastasis-to-metastasis and not a primary-to-metastasis spread.

## Discussion

Recently, molecular evolution and organismal biogeography have come into play to understand the evolutionary and spatiotemporal dynamics of cancer cells' migrations across tumors. These applications have begun to reveal insights into cancer initiation, evolution, and metastasis. Here, we have mapped terminologies between the organismal and tumor biogeography for the first time, which are needed to properly analyze tumor variation using the models and tools available in organismal biogeography. These developments bridge the gap between tumor and organismal biogeography and likely accelerate the use and advancement of existing methods.

We translated and investigated the evolutionary processes observed into a clone phylogeny through biogeographic models that weigh these processes differently. To avoid confounding the effect of clone phylogeny errors^16^ with the inaccuracy of inferences under different biogeographic models, we have used correct phylogenies in all our analyses. Even though complex models fit the data the best, simpler models reconstructed clone migration paths more accurately. This might have happened because complex models generally tend to fit better the data, but they might also infer estimates with greater variance and inaccuracy due to the low numbers of clones and/or locations, as normally sampled in real data. Another possibility is that while the +J models fit the data the best, the actual model parameters are not the same throughout a migration history, i.e., the evolutionary process is heterogeneous. In this case, estimates from even complex models can become biased, in addition to suffering from high variance.

To further understand the implication of our findings, it is important to delve into how these biogeographic approaches address and model the evolutionary processes that (clone) lineages undergo, i.e., anagenetic and cladogenetic events. Essentially, each one of the biogeographic methods studied here weighs dispersal and extinction events differently. In addition to that, models accommodate or not for genetic divergence and vicariance events.

Studies have shown that DEC and DIVA produce similar results, which agrees with our findings in tumor data analysis^17^. More specifically, the DEC model was inspired by DIVA, and so, both models assume speciation as a result of vicariance or genetic divergence. That is to say, vicariance is frequently favored over dispersal, with ancestral areas partitioned into two disjoint subsets while using the latter two biogeographic models. Furthermore, we noticed that the DEC + J and DIVA + J produced similar F1-scores regardless of the number of tumors and seeding scenario.

On the other hand, BBM and BAYAREALIKE approaches seem to favor dispersal and extinction events. We argue that BAYAREALIKE may produce more accurate clone migration inferences depending on the question at hand. These biogeographic methods are more suitable for exploring evolutionary (cladogenesis and anagenesis events) and biogeographic (dispersal, diversification, extinction, vicariance) processes simultaneously. Moreover, BAYAREALIKE offers more freedom to customize their model for exploring different tumor sizes, distances between tumors, and integrating the distance-dependent effect on the dispersal probability. Such methods will explore the size and isolation of tumors, and ultimately, tumor features and properties that are key for understanding tumor diversity and microenvironment.

Regarding the use of the *J* parameter in the biogeographic models, this is important for estimating ancestral ranges. The founder principle has its basis on the migration of a group of individuals to a remote area where they establish a small founding population. Therefore, by definition, the +J models favor dispersal and reject vicariance by predicting single areas over multiple ranges as the migration's plausible origin. The use of a +J model is important to avoid spurious estimates of the number of areas in which the ancestral clones likely existed. Without the *J* parameter, these possibilities become too large because current models allow vicariance to be a major mechanism for metastasis.

Analyses of clinical data of patients with basal-like breast cancer showed evidence of multiple clones seeding metastases, reseeding events from metastasis to primary or another metastasis, and metastasis-to-metastasis spread. The inferred seeding and source patterns for patient A1 were similar to those suggested in the original study by Hoadley et al.^13^, whereas the inferences differed for patient A7.

Our results in the empirical data analysis are further supported by the migration inferences of non-biogeographic models (MACHINA^5^ and PathFinder^7^), which indicated multiple seeding events between tumors and metastasis-to-metastasis spread. This is very interesting as previous studies using phylogenetic approaches also produced multiple clone seeding and reseeding events and propose seeding events between metastases, making apparent that the development of metastases is idiosyncratic^7,18^. Also, there is already experimental evidence of migrations between metastases in breast cancer^19–21^.

We suggest that one needs to be careful while selecting the method for real data analysis and interpreting the migration history reconstructed, even though simulation analysis suggests that methods that do not model biogeographic processes may perform better than those that do. This is because computer simulations cannot fully consider all biological attributes of metastatic migration processes. There is always a limit on the applicability of the results from computer simulations in real-life situations. Computer simulations provide useful insight into the absolute performance and relative usefulness of new methods. The results produced can be cautiously applied when different methods do not produce similar results, as seen in the analysis of the empirical datasets. More sophisticated simulations involving a larger number of clones and tumors are likely to help overcome the limitations of simulations presented here.

Furthermore, we have shown that biogeographic models could accommodate various evolutionary processes that are key when investigating the origin and trajectory of cancer cells in a patient. Models that account for founder events fit the tumor data best, however, a better fit does not translate into more accurate migration inference. In our simulation study, the most accurate approaches were the ones that do not model biogeographic processes (BBM, MACHINA, and PathFinder), but methods involving biogeographic models would be useful to estimate fundamental parameters such as the dispersal and extinction rates.

## Conclusions

In summary, we have addressed how theoretical concepts and model parametrization in various biogeographic methods capture evolutionary and biogeographic events. We discussed how the migration of cancer cells between tumors is displayed in a phylogeny. The use of concepts and principles from the field of organismal biogeography for analyzing tumor data can contribute to future applications of such methods in understanding movements and genetic diversification of cancer cells within and between tumors. Researchers have already started using biogeographic methods or developing new methods for clone migration inferences^5,8,9^. Inference of clone migration histories is a major advancement in cancer research because it enables modeling cancer cells' origin and movements between tumors. In the future, cancer researchers could harness the existent scientific knowledge from the fields of organismal evolution and biogeography to shed light on the evolution and progression of cancer disease.

## Methods

### Simulated dataset analyzed

We selected clone phylogenies and computer-simulated datasets used in previous analyses for inferring tumor migration paths; see^5,9^ for more details. Simulations varied as to the number of tumors (5–11 tumor sites; m5 and m8 datasets), the number of migrating clones (1–3), and the source-area of plausible clone migration trajectories between tumors, e.g., clones migrating between metastases or from a metastasis back to the primary tumor.

Specifically, we used 80 tumor datasets under four main clone seeding scenarios defined by the complexity of the clone migration schemes. The simplest seeding scenarios included single or multiple clones seeding from a single source-area that could be either primary or metastasis (monoclonal single-source seeding, mS and polyclonal single-source seeding, pS, respectively). The most complex seeding scenarios consisted of multiple clones seeding from multiple source-areas with primary tumor and metastasis, including reseeding events from metastasis to primary (polyclonal multi-source seeding, pM and polyclonal reseeding, pR, respectively). Essentially, these simulated datasets span a wide range of parameters/models: (a) four seeding scenarios with increasing complexity, (b) a range of the number of tumor sites (5–11), (c) single to multiple seeding events (1–3), (d) consideration of reseeding events, and a wide range of the number of (e) SNPs (9–99) and (f) clones (7–28). All relative information of clone phylogenies and tumors is available at https://github.com/raphael-group/machina/tree/master/data.

### Empirical dataset analyzed

We analyzed the dataset from the two patients with basal-like breast cancer^13^. The A1 patient consisted of nine clones from one primary tumor (breast) and four metastases (adrenal, lung, spinal, and liver) (329 SNVs). The A7 patient dataset included ten clones from primary and five metastases (kidney, liver, lung, rib, and brain) (478 SNVs)^13^. We used the clone phylogeny from the original study, which was rooted using the germline sequences (normal cells). We assumed clones were different when they were sampled in more than one tumor site. We did this to reduce the inference of multiple ranges at ancestral nodes, a common issue for biogeographic methods that consider vicariance over dispersal to explain migration events.

### Biogeographic analyses

We used six biogeographic models, each one treating evolutionary processes differently (Table 1). We chose six models implemented in the BioGeoBEARS R package^14^. We used the default implementation of BioGeoBEARS, which is Maximum Likelihood (ML) of the following methods: BayArea^22^, Dispersal Extinction Cladogenesis (DEC)^23^, and Dispersal-vicariance analysis (DIVA)^24^. We also added a parameter that accounts for the founder-event speciation in each method mentioned above (*J*). Thus, six biogeographic models implemented in BioGeoBEARS were used: BAYAREALIKE, BAYAREALIKE + J, DEC, DEC + J, DIVALIKE, and DIVALIKE + J (Table 1). Also, we used the Bayesian Binary MCMC (BBM) method implemented in RASP v4, which has been explored previously and shown to infer relatively reliable clone migrations^9,25,26^.

We used the true clone phylogenies and tumors as the sampled clones' location because we were interested in examining the effect of various biogeographic processes on the accuracy of clone migration inferences. True phylogenies were derived from the original study and reproduced using the maximum parsimony (MP) method in MEGA-CC because simulated sequences did not have any homoplasy^9,27^. The outgroup was a sequence with no mutations. All models implemented in BioGeoBEARS and BBM were given a topology that contained branch length information. Essentially, the first input file to the BioGeoBEARS and BBM is a clone phylogeny, which the user may have inferred from tumor sequencing data using available computational methods. The second input file is a list of locations where each clone is found (Fig. 2).

We analyzed clone phylogenies under six biogeographic models in the R package BioGeoBEARS. These analyses produce probabilistic inferences of geographic ranges on ancestral nodes of a phylogeny by considering different models of geographic range evolution. The generated output files include (1) the predicted ancestral location at each node and (2) the probability value for the prediction. Next, we manually reconstructed the migration paths (Fig. 2).

BioGeoBEARS uses LAGRANGE that models two free parameters: dispersal rate, i.e., the rate of range expansion by adding an area along a phylogenetic branch (*d*), and extinction rate, i.e., the rate of local range loss through along a phylogenetic branch (*e*)^17,23^. BioGeoBEARS also allows for modeling founder-event speciation (*J*) in which the new species jumps to a range outside of the ancestral range. The addition of the *J* parameter results in models with three parameters. Each of the six models we used in our analyses had its implementation. Notably, BAYAREALIKE and BAYAREALIKE + J modeled dispersal and extinction. On the other hand, DEC, DEC + J, DIVALIKE, and DIVALIKE + J models integrated a fixed cladogenetic model that gives an equal probability to vicariance and extinction events (Table 1).

For the six biogeographic models implemented in BioGeoBEARS, we removed the null range from the state space such that the sampled clones were required to be present in at least their sampling location. The null range would have accounted for the possibility of a clone being present in no areas, meaning that it was not sampled in any of the sampled tumors. The ancestral nodes had a maximum range of size two, which means that a maximum of two different tumor sites was allowed to be assigned as a possible ancestral range area. We did that to avoid erroneous migration inferences because of the multiple ranges suggested at ancestral nodes.

Instead of using ultrametric trees, which is the ideal input for BioGeoBEARS, we provided an ultrametric-like format for our data analysis as suggested by the author of BioGeoBEARS (personal communication with Dr. Nicholas Matzke). The unit for the branch lengths in the ultrametric trees is absolute or relative time. In clone phylogenies, we only have branch lengths in the unit of the number of mutations per site. So, we used the number of mutations as a proxy for time, which we called the ultrametric-like tree. Tip clones connected with zero branch length were considered ancestral; they were without recent diversification events. We used the default settings for the other parameter settings (see https://github.com/nmatz0ke/BioGeoBEARS). An area with the highest inference probability at an internal node of the phylogeny was considered the clone’s ancestral range. Migration paths were obtained when the reconstructed ranges differed between an internal node and its direct descendant node.

The analyses for the BBM approach were run in RASP^26^. BBM method uses a full hierarchical Bayesian approach for inferring ancestral states. The RASP implementation allows for annotating dispersal and extinction events to the ancestral distributions and their probabilities at each node. We obtained the clone migration inferences by BBM from Chroni et al.^9^. The analysis settings included three runs of the Markov-chain Monte Carlo chains for 5,000,000 generations to ensure that MCMC chains would reach stationarity and convergence. The stationary rate frequencies and reconstructed states were sampled every 1000 generations, and there was a 10% burn-in. All analyses were run under the fixed Jukes-Cantor (JC69) with equal character (tumor site) change (dispersal) rates (for more details about the criteria of the settings for BBM, see^9^). Runs were combined into a single result, which we used for evaluating the inferred clone migration paths.

### Accuracy measurements

The accuracy of the migration paths reconstructed for the tumor-simulated data was evaluated through F1-scores. These indicate a method's accuracy by counting true positives (TPs), false positives (FPs), and false negatives (FNs) results. F1-scores are the harmonic mean of precision and recall:

$${\text{F}}1 = 2 \times \frac {{\text{precision}} \times {\text{recall}}} {{\text{precision}} + {\text{recall}}}$$

where

$$\text{precision} (\text{G}, \text{G}^{*}) = \frac { {\text{TP}} } {{\text{TP}} + {\text{FP}}}$$

and

$$\text{recall} (\text{G}, \text{G}^{*}) = \frac {{\text{TP}}} {{\text{TP}} + {\text{FN}}}$$

For each dataset, one clone migration history was inferred under each biogeographic model. We identified clone migration paths as correct (TPs), erroneous (FPs), and not-inferred (FNs).
